# Oxygen producing microscale spheres affect cell survival in conditions of oxygen-glucose deprivation in a cell specific manner: implications for cell transplantation†
†Electronic supplementary information (ESI) available. See DOI: 10.1039/c8bm00490k


**DOI:** 10.1039/c8bm00490k

**Published:** 2018-08-22

**Authors:** Heike Newland, Dimitri Eigel, Anne E. Rosser, Carsten Werner, Ben Newland

**Affiliations:** a Leibniz Institute of Polymer Research Dresden (IPF) , Hohe Strasse 6 , 01069 Dresden , Germany; b Brain Repair Group , School of Biosciences , Cardiff University , CF10 3AX , UK; c School of Pharmacy and Pharmaceutical Sciences , Cardiff University , CF10 3NB , UK . Email: newlandb@cardiff.ac.uk

## Abstract

This study outlines the synthesis of microscale oxygen producing spheres, which, when used in conjunction with catalase, can raise the dissolved oxygen content of cell culture media for 16–20 hours. In conditions of oxygen and glucose deprivation, designed to mimic the graft environment *in vivo*, the spheres rescue SH-SY5Y cells and meschymal stem cells, showing that oxygen producing biomaterials may hold potential to improve the survival of cells post-transplantation.

## Abstract 2

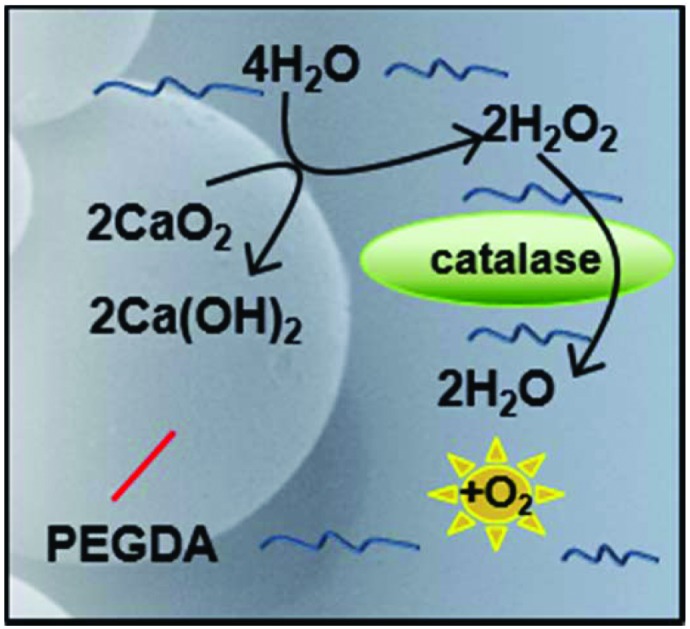
Oxygen-glucose deprivation detrimentally affected mesenchymal stem cells, which could be reversed by the addition of oxygen producing spheres.

## 

Cell death post transplantation is probably a multifactorial issue, however, analysis of grafts immediately post transplantation to the brain revealed that extreme hypoxic conditions within the graft (especially the core) coincided with apoptosis and death of the graft.^1^,^2^ This data may indicate that a lack of oxygen in the graft may partially be to blame for the rapid cell loss post transplantation.

One strategy for improving cerebral transplant survival may therefore be to promote angiogenesis at the graft site, which should improve the flow of oxygen and nutrients to the grafted cells. In line with this strategy, researchers have developed pharmacologically active microcarriers which release vascular endothelial growth factor (VEGF) to the grafted cells in order to increase angiogenesis at the graft site.^3^ Another strategy, would be to provide oxygen to the grafted cells for the early period post transplantation until vascularization of the graft has occurred.^4^

Limited nutrient supply to the graft core is likely to affect different cell types in different ways. We synthesized oxygen producing microspheres and analyzed the effect these had on the viability of three cell types cultured in conditions designed to mimic deprivation of vital nutrients (oxygen and glucose). The synthesis of biomaterials that produce or release oxygen is a research field that has received much interest.^5^ Materials that carry and release oxygen, such as fluorinated oils and hemoglobin based oxygen carriers, have long been studied, especially in the context of blood substitues.^6^ Biomaterials that produce oxygen require an oxygen source such as sodium percarbonate,^7^ calcium peroxide^8^–^11^ or sodium peroxide.^12^ Upon contact with water, and/or catalase, these oxygen sources break down to yield molecular oxygen and water. Typically these are embedded in a surrounding polymer matrix, such as poly(lactic-*co*-glycolic acid) (PLGA),^7^,^8^,^12^ or polydimethylsiloxane (PDMS)^9^ to prolong the oxygen delivery time. These oxygen producing biomaterials have taken a variety of forms, including bulk hydrogels,^10^ a sphere/hydrogel mix,^13^ scaffolds,^8^ films^7^ and disks.^9^ For applications where injection is necessary, the oxygen producing materials should be smaller than the internal diameter of the needle. To this end, various emulsions have been used to create microparticles/microspheres capable of producing oxygen.^14^,^15^ Transplantation to the rodent brain typically requires 30G needles/cannula with an internal diameter of 160 μm, so we set out to produce simple and scalable microspheres under 100 μm diameter that were capable of releasing oxygen. It was hypothesized that modifications of the dual-photoinitiator, water in oil emulsion-based technique developed by Franco, C. L., *et al.*^16^ could be used to produce high numbers of poly(ethylene glycol) diacrylate (PEGDA) based oxygen producing spheres. Furthermore, we aimed to analyze whether these spheres could rescue three different cell types from the detrimental effects of oxygen and glucose deprivation (harsh conditions imposed in order to simulate cell transplantation to the brain).

Spheres consisting of PEGDA and calcium peroxide were synthesized in the absence of solvent by mixing these two components with Pluronic F68 (non-ionic surfactant) and 2-hydroxy-2-methylpropiophenone as a photoinitiator (see Fig. 1 for a schematic depiction of the process). By vortexing this mixture within mineral oil (containing Span 85 and Tween and a second photoinitiator (IRGACURE 651)) an emulsion was formed which was then exposed to UV light to crosslink the PEGDA (see ESI† for detailed synthesis procedure). Using different photoinitiators in each phase allows better shaped microsphere formation as shown previously.^16^,^17^ Scanning electron microscopy (SEM) revealed well-formed, individual spheres had been produced (Fig. 1c, ESI Fig. S1 and S2†). The addition of Pluronic F68 to the reaction mixture vastly reduced the size distribution of the spheres and improved the oxygen release profile, so was adopted for all subsequent sphere formation (ESI Fig. S2c and S2f†).

**Fig. 1:**
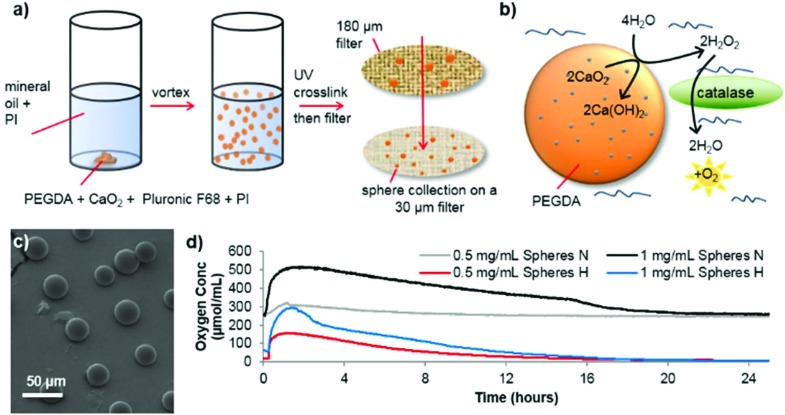
Schematic depiction of the PEGDA/CaO_2_ microsphere synthesis process (a) using the dual photoinitiator (PI) approach. Oxygen production is shown in (b) whereby contact of the sphere with water produces hydrogen peroxide. The presence of catalase in the surrounding media ensures the rapid break down of hydrogen peroxide to water and oxygen. Scanning electron microscopy (SEM) image of the oxygen producing spheres (c), and the corresponding oxygen release profiles (d) of different concentrations of spheres in normoxic conditions (N) or hypoxic conditions (H).

As Fig. 1b depicts, to start of the oxygen release process, contact with water is required. The microsphere production procedure, in anhydrous conditions, allows dry spheres to be harvested which retain their potential activity during storage in dry conditions (see ESI Fig. S3†). Once water has penetrated the PEG network to contact the CaO_2_, it is converted to hydrogen peroxide. As expected, this reaction intermediate is highly cytotoxic, which is shown in ESI Fig. S4† *via* the use of human SH-SY5Y cells (chosen as a model dopaminergic cell^18^), which were cultured *in vitro* in two conditions; normoxia (21% O_2_) and extreme hypoxia (0.1% O_2_). However, adding catalase to catalyze the break down process of hydrogen peroxide completely reverses this loss in cell viability. Analysis of the oxygen release from the spheres (OXY-4 mini non-invasive oxygen sensor, PreSens, Germany), showed that an elevated oxygen concentration lasts up to 16 hours in both normoxic and hypoxic conditions (see Fig. 1d). Similar release profiles can be seen for both conditions though a faster rate of oxygen decrease occurs in hypoxic conditions.

In order to analyze the spheres in cell culture, it was first desired to analyze the oxygen consumption rate of cells in a closed system (*i.e.* no gaseous exchange) with or without the oxygen producing spheres. Although no transplantation situation would ever resemble a completely closed system (however poor the surrounding vasculature is, there would likely be some minimal amount of oxygen diffusion), it would provide an idea of how quickly the oxygen is consumed from the transplantation media. Three different cell types were chosen for the following analyses. As mentioned previously, SH-SY5Y cells are commonly used as a model dopaminergic cell,^18^ so these were used as a testing platform herein. Primary embryonic ventral mesencephalic (VM) cells are commonly used as a source of dopaminergic neurons for cell transplantation to Parkinsonian animal models^19^ and to Parkinson's disease patients in the TRANSEURO clinical trial.^20^ In addition, mesenchymal stem cells (MSCs) produce a potentially neuroprotective secretome,^21^ so they have also been investigated for cell transplantation to the Parkinsonian brain.^22^,^23^ To analyse the oxygen consumption rate of these three cell types, 500 000 cells (a typical number of grafted cells per brain hemisphere^19^) were seeded in 350 μL of media into the well of a 96-well plate equipped with the PreSens non-invasive oxygen probe. A second group of cells were seeded in media containing the oxygen containing spheres and catalase. The plate was immediately sealed with a plate sealant and the dissolved oxygen content measured every minutes. Fig. 2 shows that the highly proliferative SH-SY5Y cells rapidly consumed oxygen, almost depleting all the oxygen in the sealed chamber by 48 hours. In contrast, the VM cells used comparatively little oxygen which stabilized at a level of ∼100 μmol mL^–1^ after 24 hours. The MSC consumption was comparable to that of the SH-SY5Y cells, with a continuing downward trend being shown up to 48 hours. For all cases, the addition of oxygen producing spheres created an initial increase in oxygen level that eventually came down to near that of the cells seeded in media alone. Since this experiment was designed to analyse the oxygen consumption rate of a typical graft, it has a major limitation in that the media volume was far too large. Whilst transplantation into rodents typically requires a transplantation volume of up to 4 μL, this experiment used 350 μL, purely due to limitations of the probe/chamber size. It does, however, show empirically that different cell types have vastly different oxygen consumption rates, and one would expect complete depletion of oxygen to occur much faster within a 4 μL graft.

**Fig. 2:**
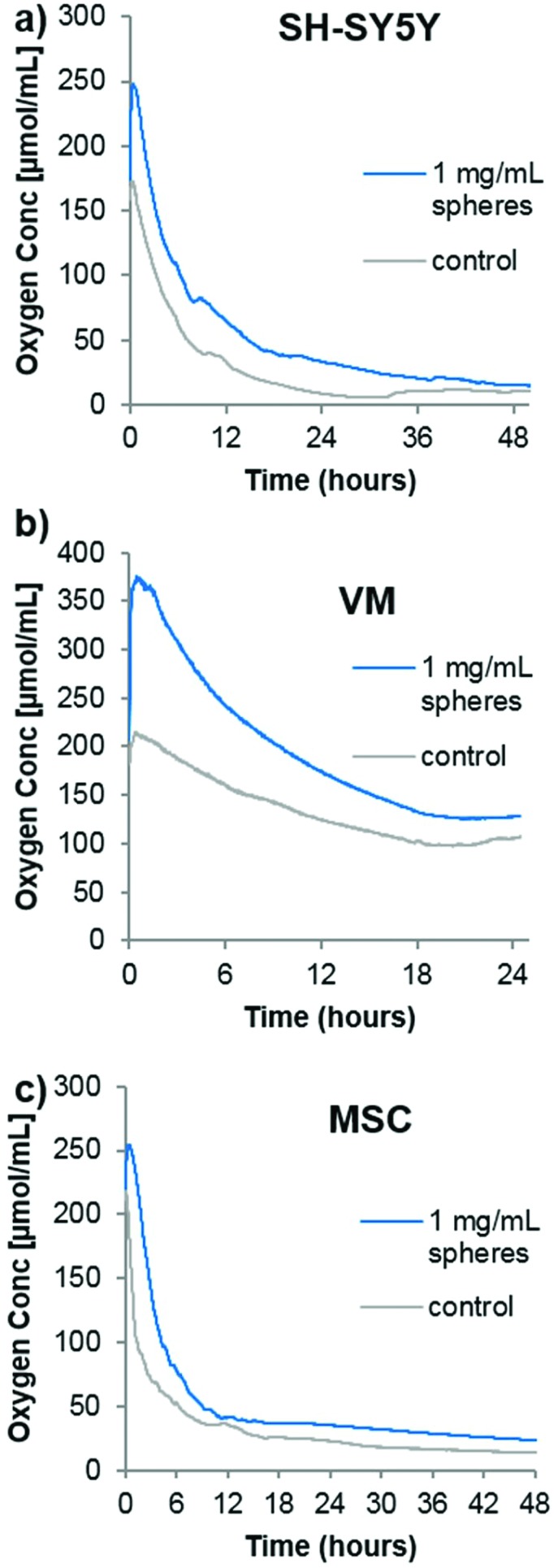
Oxygen producing spheres compensate for cellular oxygen consumption. Graphs showing the oxygen consumption of 500 000 SH-SY5Y cells (a), ventral mesencephalic cells (VM) (b) or mesenchymal stem cells (c) seeded in a sealed chamber either without spheres (grey line) or with 1 mg mL^–1^ oxygen producing spheres (blue line) (*n* = 2, error bars omitted for clarity).

Praet *et al*. have shown that hypoxia in grafts can clearly be measured as early as six hours post transplantation, and cell death occurs prior to neoangiogenesis (observed at three days post transplantation).^2^ Taken together, there is most likely a window of opportunity where oxygen producing materials could improve survival before adequate angiogenesis has occurred. Depletion of oxygen may, however, be only part of the detrimental effect to the graft core. Poor diffusion of other nutrients, such as glucose, to the graft core may also be playing a role in the cell death. To this end, we investigated the role of both oxygen and glucose simultaneously by culturing the three cell types in oxygen – glucose deprivation (OGD) conditions and measuring their metabolic activity (used as a measure of viability by normalization to control cells) as an indicator of cell health. Fig. 3 shows that by the second day of culture, SH-SY5Y cells were slightly effected by hypoxic conditions, which was exacerbated by low glucose. The cell viability of VM cells was not affected by extreme hypoxia or low glucose levels. Remarkably this situation persisted even to five days of culture time (data not shown). However, MSCs cultured in low glucose for two, three and four days showed a reduced cell viability, and hypoxic conditions further reduced this viability after three and four days in culture.

**Fig. 3:**
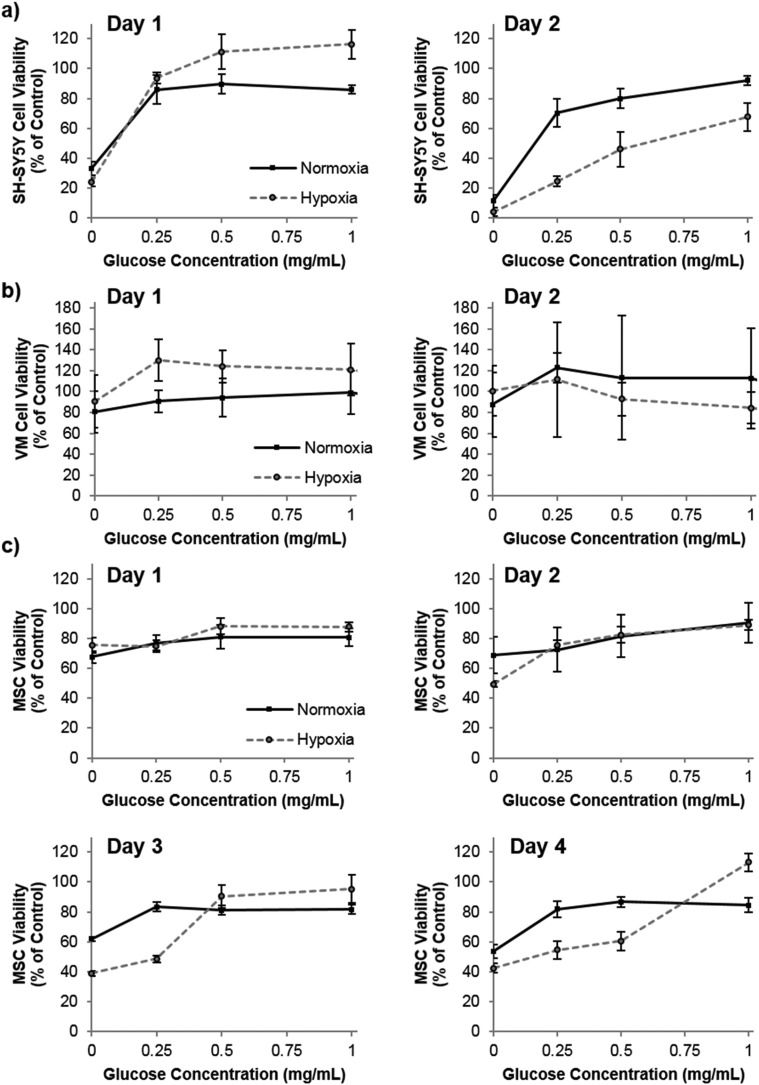
The effect of oxygen-glucose deprivation (OGD) on cell viability. SH-SY5Y cells (a), VM cells (b) and MSCs (c) were cultured in 0.1% oxygen (hypoxia – dashed lines) or 21% oxygen (normoxia – black lines) in varying concentrations of glucose. Low glucose and low oxygen was detrimental to the cell health of SH-SY5Y cells and MSCs, *n* = 4 error bars represent ± standard deviation.

Our studies corroborate the findings of Deschepper *et al.*, which shown that MSCs are capable of surviving 21 days in hypoxic conditions but die if deprived of both oxygen and glucose simultaneously.^24^ The authors show that this is due to a switch to anaerobic metabolism in near anoxic conditions. Whilst it was clear for SH-SY5Y cells that glucose is playing a more important role in cell health than oxygen in our experimental set up, there was also a clear difference in viability between MSCs cultured in low glucose normoxia and those cultured in low glucose hypoxia (OGD). Theoretically therefore, one could envisage that the oxygen producing spheres could rescue MSCs from the detrimental effects of OGD. Fig. 4 shows how the addition of oxygen producing spheres to the cell media of cells cultured in OGD conditions affects cell viability.

**Fig. 4:**
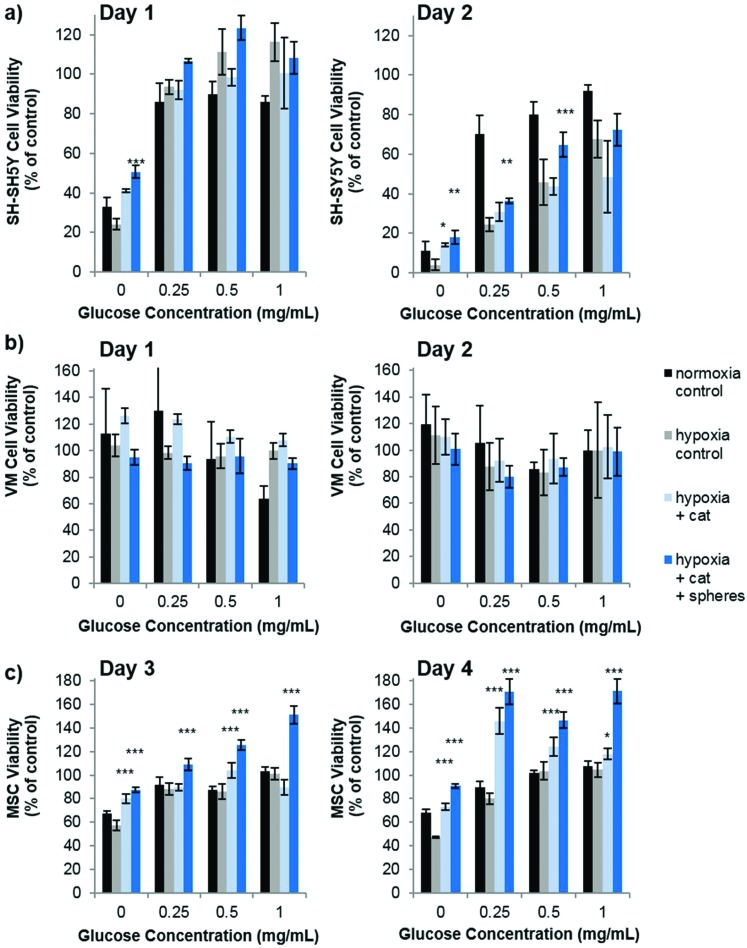
Oxygen producing spheres improve the viability of SH-SY5Y cells and MSCs cultured in conditions of oxygen-glucose deprivation (OGD). After two days of OGD culture SH-SY5Y cells show reduced viability compared to those cultured in normoxia which can be reversed by the addition of catalase and oxygen producing spheres (a). OGD culture has no effect of VM cell culture so no rescue effect was observed through the addition of oxygen producing spheres (b). MSCs show a loss of viability in OGD conditions which is most pronounced after four days in culture (c). Addition of catalase and oxygen producing spheres results in a dramatic increase in cell viability. For all studies *n* = 4, error bars represent ± standard deviation, asterisks represent a statistical significant difference from hypoxia control (two-way ANOVA where *p < 0.05, **p < 0.01., ***p < 0.001).

As predicted, addition of the oxygen producing spheres has no effect on VM cell viability since the deprivation of oxygen does not cause a loss in viability (Fig. 4b). However, on the second day of culture, SH-SY5Y cells cultured in hypoxia with 0, 0.25 and 0.5 mg mL^–1^ glucose show a small but significant improvement in viability in the presence of 1 mg mL^–1^ oxygen producing spheres and catalase (Fig. 4a). Larger improvements in viability can be observed for MSCs cultured in hypoxic conditions with oxygen producing spheres compared to those without (Fig. 4c). Two other phenomena arise, the first is that catalase alone (*i.e.* used without the oxygen producing spheres but at the same concentration) resulted in increased viability of the MSCs at many of the different glucose concentrations. The second is that MSCs cultured with catalase and the oxygen producing spheres together sometimes had higher viability than the cells cultured in normoxic conditions (normoxia control on the graphs). The first may be explained by the fact that MSCs cultured in hypoxic conditions exhibit increased hydrogen peroxide production which can contribute to cell death.^25^ The addition of catalase therefore reduces the level of hydrogen peroxide and other reactive oxygen species allowing better viability. The second phenomena of higher cell viability in the catalase plus oxygen producing spheres group is harder to explain, but may be due to a combinatorial effect of increased oxygen plus the presence of catalase, which could have resulted in higher glucose metabolic activity rates. It can, however, be concluded that in these *in vitro* OGD culture conditions, the addition of the oxygen producing spheres has stronger beneficial effects on MSC viability than SH-SY5Y cells and no effect is seen for VM cells. It should be highlighted here that the medium used for the OGD experiments contained 10% FBS which contained 0.509 mg mL^–1^ of glucose. There was therefore an additional final glucose concentration of 0.05 mg mL^–1^ within all glucose concentrations tested.

Praet *et al.*, show that only three days after embryonic fibroblasts are grafted to the mouse brain (right capsula externa) neoangiogenesis within the graft is taking place.^2^ In this case the rapid vascularization may be aided by the grafted cells innate ability to produce VEGF. However, regardless of the cell type being transplanted to the brain, there will be a window of delay between grafting and vascularization of the graft. This window may be in the order of days and weeks (until anastomosed blood vessels formed), but a major limitation of the oxygen producing materials developed herein is that they could only provide a source of oxygen for the first 16–20 hours post transplantation. It is therefore clear, that whilst the data in Fig. 4 shows sphere mediated rescue occurs *in vitro*, better materials need to be prepared that release oxygen over longer time periods for application *in vivo*. PEGDA, crosslinked in anhydrous conditions was chosen as the means to encapsulate the calcium peroxide and contain the solid breakdown components. In this way the spheres could be prepared in the absence of water and obtained as a dry powder ready for use. Poly(ethylene glycol) has often been used as a component of biomaterials used in the brain without dramatically elevated host response,^26^–^29^ so was considered a valid starting material. However, future studies will focus on more hydrophobic polymers to restrict the access of water to the calcium peroxide. A range of candidate monomers will be used such as the more hydrophobic ethylene glycol dimethacrylate, di(trimethylolpropane) tetraacrylate and bisphenol A ethoxylate diacrylate in order to assess whether the oxygen release time can be increased. In addition, other factors need to be taken into consideration such the method of catalase delivery, and the host response to the material. In this study catalase was used in solution (in the media) but future studies may consider whether it should be tethered to the sphere.^14^ A clear limitation of this work is the lack *in vivo* analysis. These studies have attempted to create an *in vitro* model that mimics transplantation conditions in order to assess new oxygen producing biomaterials. Though it may serve good use in initial studies, extrapolation to the *in vivo* condition is tenuous and would need validating with experimental study.

Our data seem to suggest that oxygen producing materials may improve the survival of grafts of MSCs but not would assist fetal dopamine cells of the ventral mesencephalon during the transplantation process. Furthermore, it should be noted that whilst the presence or absence of oxygen producing spheres effected SH-SY5Y and MSC viability, the concentration of glucose in the media appeared to play a much larger role. This data corroborates the recent findings that glucose is the major determinant of MSC viability in near anoxic conditions.^30^

## Conclusions

Taken together these experiments present the synthesis of microscale spheres comprised of calcium peroxide encapsulated in crosslinked poly(ethylene glycol) diacrylate. Once placed into a solution containing catalase, oxygen is produced for up to 16 hours. In an attempt to mimic a transplantation scenario, we show that SH-SY5Y cells and MSCs consume oxygen more rapidly than VM cells and that they are more sensitive to culture in OGD conditions. Addition of the oxygen producing spheres in media containing catalase reverses the detrimental effect of OGD. This data suggests that co-injecting oxygen producing materials during cell transplantation may an effective means to improve cell survival. Future studies should focus on longer term oxygen release, so as to provide oxygen to the grafted cells before sufficient vascularization of the graft has occurred.

## Conflicts of interest

The authors declare no conflicts of interest.

## Supplementary Material

Supplementary information
