# Supplementary material for: Oxygen producing microscale spheres affect cell survival in conditions of oxygen-glucose deprivation in a cell specific manner: implications for cell transplantation
Source: Biomater Sci. 2018 Aug 22;6(10):2571–7. doi: 10.1039/c8bm00490k (PMC6157640; doi:10.1039/c8bm00490k)
Supplement: Supplementary file 1 [file BM-006-C8BM00490K-s001.pdf]

Electronic Supplementary Material (ESI) for Biomaterials Science.
This journal is © The Royal Society of Chemistry 2018

Supporting Information

Oxygen producing microscale spheres affect cell survival in conditions of oxygen-glucose deprivation in a cell specific manner: implications for cell transplantation

Heike Newland, Dimitri Eigel, Anne E Rosser, Carsten Werner, Ben Newland

Materials and Methods

Materials

Polyethylene (glycol) diacrylate (PEGDA) (Mn 700 g/mol) and the reactive oxygen source calcium peroxide (Mw: 72.08 g/mol) were purchased from Sigma. To initiate the photopolymerisation the highly efficient photoinitiator IRGACURE® 651 (Ciba®) was used in combination with 2-hydroxy-2-methylpropiophenone (Sigma). 10% Pluronic® F 68 (gibco; Life Technologies) was used as an emulsifier. The oil phase consisted of mineral oil (PlusOne Drystrip Cover Fluid) supplemented with 1 % of the non-ionic detergent sorbitane trioleate (SPAN® 85), and 2 % of polyethylene glycol sorbitan monolaurate (TWEEN® 20). Catalase from bovine liver (2000-5000 units/mg) purchased from Sigma, filtered through a 0.2 μm filter, and lyophilized before use.

Synthesis of oxygen producing spheres

An adaptation of the previously published dual-photoinitiator, emulsion-based technique was used to form oxygen releasing microbeads.[1] 20 μL of the liquid PEGDA monomer containing one percent (vol/vol) 2-hydroxy-2-methylpropiophenone and 20 percent calcium peroxide (wt/vol) was mixed with 10 μL Pluronic F68. This suspension was added to 1 mL of mineral oil containing 1 % Span 85, 2 % Tween and 0.5 % IRGACURE® 651 then vortexed (Vortex Genie2, Scientific Industries) for 30 seconds at full speed. The emulsion was immediately exposed to UV light (315-500 nm) for one min (Delolux 04, 8 Watt/cm^2) to initiate the photopolymerisation. The spheres were washed five times with Tetrahydrofuran (THF), centrifuging at 13,000 rpm for 30 seconds in between each wash, to pellet the spheres. They were then filtered through a 180 μm nylon net filter (Merck Millipore Ltd.) followed by bead collection on a 30 μm nylon net filter.

Microsphere characterization

To determine the size distribution of the oxygen producing spheres after filtration, images were taken using a light microscope with 20x objective lens (Olympus IX73) and analysed using ImageJ processing software. A minimum of 75 spheres were measured for each condition (either those containing Pluronic, or those without). The surface properties of the spheres were analyzed by scanning electron microscopy (SEM). The spheres were dried on carbon coated SEM stubs before being sputter coated with gold (BALZERS SCD 050 Sputter Coater, Germany) for 60 seconds. Images were taken using a XL30 ESEM-FEG microscope (Philips, Netherlands) in high vacuum mode using accelerating voltages of 5 kV.

Oxygen release analysis

The oxygen release profiles were measured via a fiber optic oxygen transmitter (OXY-4mini PreSens). Spheres were re-suspended at a concentration of 0.5 mg/mL or 1 mg/mL in 300 μL of PBS containing 0.5 mg/mL catalase. These were then added to measuring cuvettes in either

normoxic (21% oxygen) or hypoxic (0.1% oxygen)(Biospherix hypoxia C-chamber) conditions at 37°C. Experiments were performed in duplicate and measurements were taken every minute.

Cell culture

For cell culture experiments the human neuroblastoma cell line SH-SY5Y (Sigma) between passages 17-35, primary ventral mesencephalic (VM) cells from the midbrain of embryonic rats (E12-14) and human mesenchymal stem cells (MSC) were used. The primary VM cells were a kind gift from Prof. Storch of the University Hospital Dresden. SH-SY5Y cells were cultured in Dulbecco´s Modified Eagle´s Medium with nutrient mixture F-12 (DMEM/F-12) and GlutaMAX™, supplemented with 10 % (v/v) heat-inactivated fetal bovine serum (FBS), 1% penicillin [100 U/mL] and streptomycin [100 µg/mL]. VM cells were cultured in high glucose DMEM and supplemented with 10% FBS, 1% Pen/Strep and 10 mM HEPES buffer on poly-D-lysine (PDL, Sigma) coated surfaces. MSCs were kindly provided by the laboratory of Professor Martin Bornhäuser which were isolated from male human bone marrow aspirates (donor age 20-40 years) (University Hospital Carl Gustav Carus, Technische Universität Dresden) as previously described.[2] Cells were maintained in humidified atmosphere of 5% CO_2 at 37°C.

Effect of oxygen producing spheres on cell viability

The impact of the oxygen producing spheres on SH-SY5Y cell viability was analysed both in the presence and absence of catalase (0.5 mg/mL) using the PrestoBlue® cell viability reagent (Thermo Fisher Scientific). 10 % PrestoBlue® was added to culture media followed by half an hour incubation at 37°C. The fluorescence signal was measured using a Tecan GENios plate reader with an excitation wavelength of 540 nm and an emission wavelength of 550 nm. PrestoBlue® directly measures cell metabolic activity, so these values were was normalized to that of untreated cells (termed 100% viability) to give a measure of cell viability. To ensure that the materials were not influencing the assay the following blank controls (no cells) were assessed and found to have no effect on the fluorescent readout: PrestoBlue® in media, PrestoBlue® with spheres at 1 mg/mL, PrestoBlue® with catalase at 0.5 mg/mL, and PrestoBlue® with spheres and catalase (aforementioned concentrations). All experiments were performed in quadruplicate.

Oxygen consumption assay

500,000 cells (SH-SY5Y and VM) or 475,000 cells (MSC) were seeded per well into a wells of a 96-well place fitted with the PreSens non-invasive oxygen detection spots (PreSens, Germany). A total media volume of 350 µL was used per well either with, or without, oxygen producing spheres (1 mg/mL spheres, 0.5 mg/mL catalase). The plate was then sealed using adhesive foils for qPCR (Genaxxon). The dissolved oxygen content was analyzed every minute for 48 hours via optical cables directed towards the spot sensor.

Effect of oxygen – glucose deprivation (OGD) on cell viability

SH-SY5Y cells and MSCs were seeded at a density of 10,000 cells/well, and VM cells were seeded at 50,000 cells/well) in 96 well-plates (PDL coated for VM cells) and left overnight to adhere in normoxic conditions. The next day, the media was removed and replaced with media containing different concentrations of glucose from 0 to 1 mg/mL and placed in either normoxic (21% oxygen) or hypoxic (0.1% oxygen) conditions. For all of these experiments, regardless of cell type, glucose free DMEM (Gibco) was used with 10% FBS (Biochrom, Cat# S 0015) and glucose (Sigma) was added to the correct concentration and sterile filtered.

Prior to the media change, the media for hypoxic conditions had been placed into the hypoxia chamber for a minimum of four hours to remove dissolved oxygen. After the desired incubation periods the PrestoBlue® assay was performed to analyze cell viability. All experiments were performed in quadruplicate.

Analysis of rescuing effect of oxygen producing spheres on cells in OGD culture

The above experiment was repeated but with four experimental groups: cells alone in normoxia, cells alone in hypoxia, cells + catalase in hypoxia, cells + catalase + spheres in hypoxia. The final concentration of catalase was 0.5 mg/mL and the final concentration of spheres was 1 mg/mL to match previous experiments. After the desired incubation periods the PrestoBlue® assay was performed to analyze cell viability. All experiments were performed in quadruplicate.

Statistical Analysis

Statistical analysis was performed using Graph Pad Prism software using a two way ANOVA to distinguish statistically significant differences above the hypoxia control group in Figure 4.

Supporting Information Figures

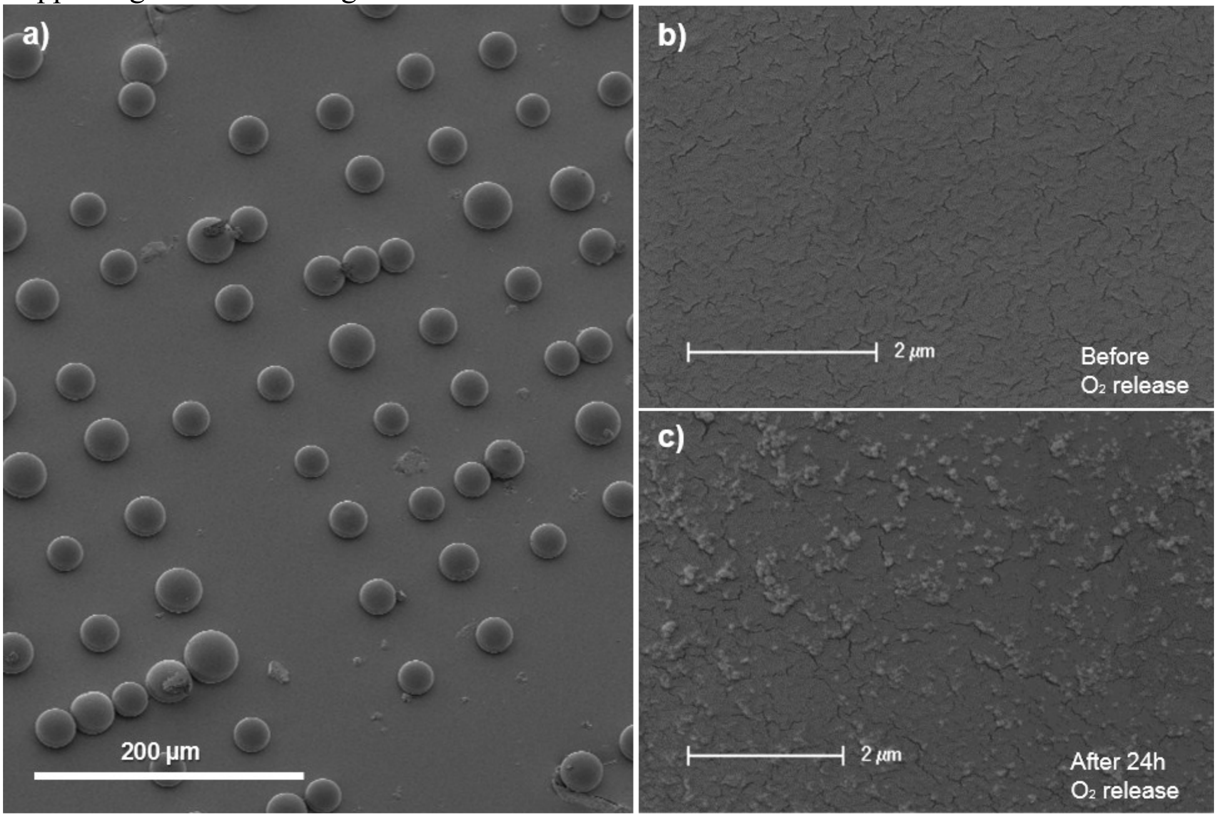

SI Figure S1 *- Scanning electron microscopy of* ***(a)*** *the oxygen producing spheres, with a higher magnification images of a sphere surface before* ***(b)*** *and after 24 hours oxygen release* ***(c)***.

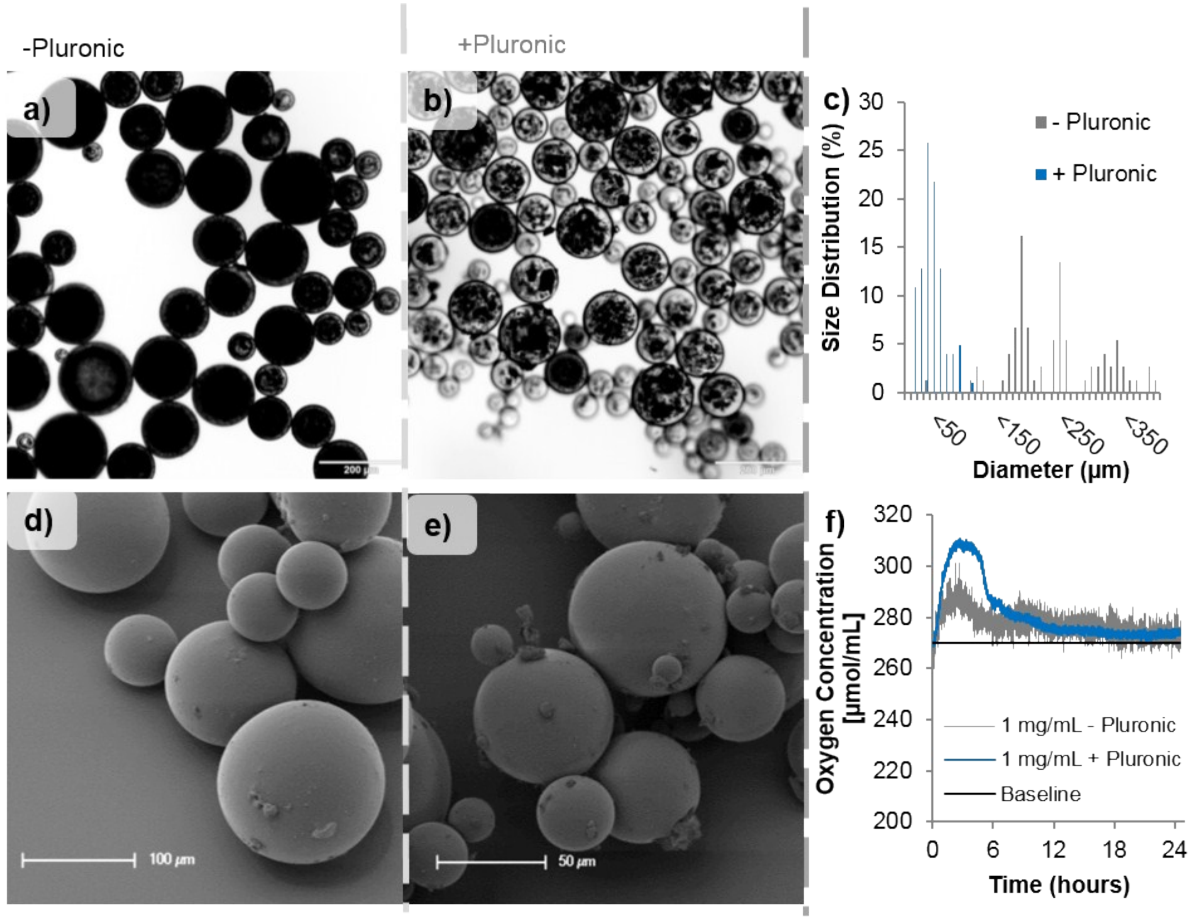

SI Figure S2 *- The effect of Pluronic® F 68 surfactant on sphere characteristics. Oxygen producing spheres synthesized in the absence* ***(a, d)*** *and presence* ***(b,e)*** *as imaged by light microscopy* ***(a,b)*** *and scanning electron microscopy (d,e). A high impact of pluronic is noticeable on the percentage size distribution of the spheres* ***(c)*** *(n=80) as well as at the oxygen release profiles* ***(f)****.*

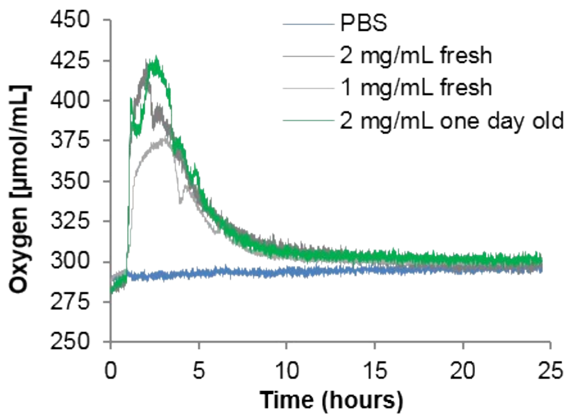

SI Figure S3 - *The oxygen release profile of different concentrations of freshly prepared oxygen producing spheres in comparison to spheres that had been stored in their dry state at room temperature for 24 hours.*

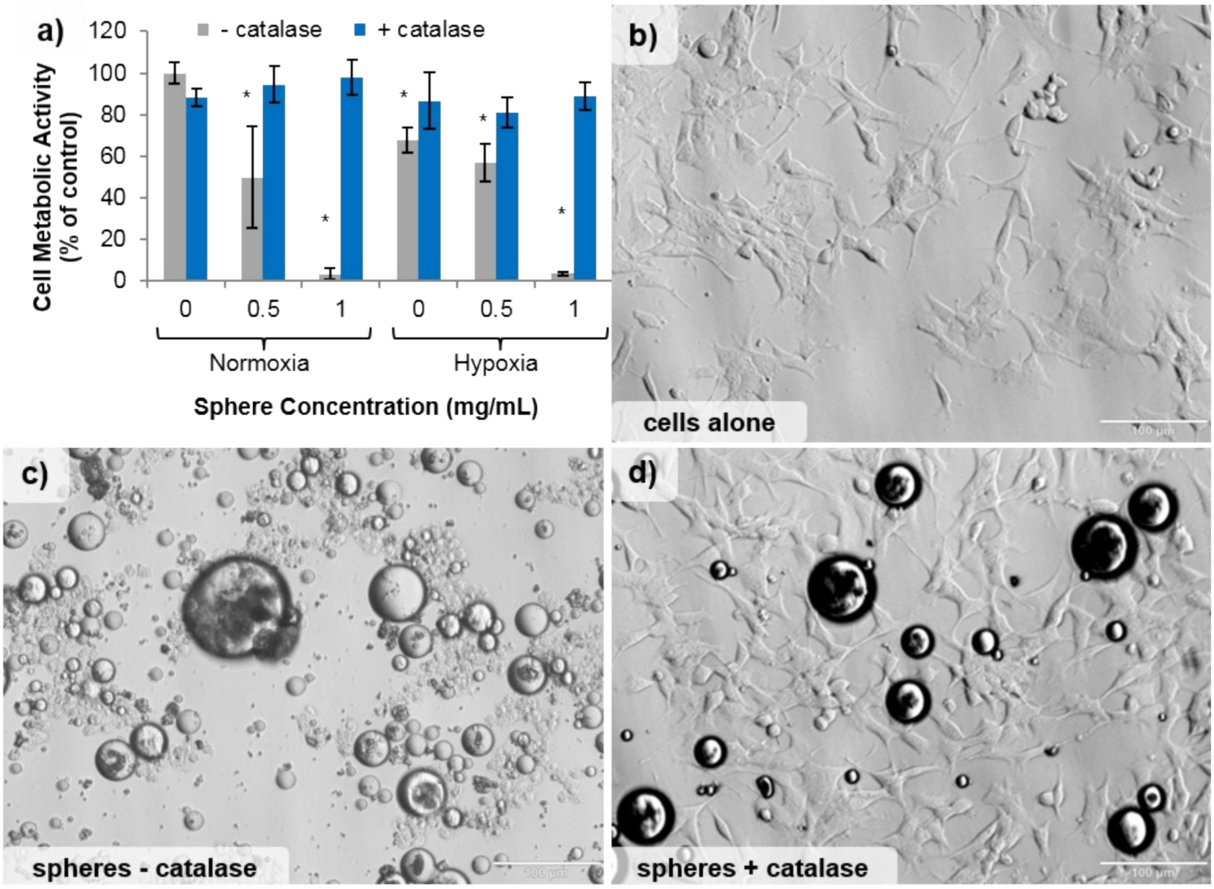

SI Figure S4 *- Characterization of the cellular viability and oxygen concentration in the presence of the oxygen producing spheres. SH-SY5Y cells were cultured either in normoxic (21% O_2) or in extreme hypoxic (0.1% O_2) conditions and the oxygen producing microspheres were added either in the presence or absence of catalase in the media.* ***(a)*** *Analysis of the cell viability (PrestoBlue assay) after 1 day in culture showed that the spheres cause no significant toxicity in the presence of catalase but reduce viability as expected when no catalase is used (n=4, error bars represent +/- standard deviation and * represents a statistically significant difference (P < 0.5 – one way ANOVA) to the control group (cells cultured in normoxia without spheres and without catalase). Panels* ***(b)****,* ***(c)*** *and* ***(d)*** *show the corresponding light microscopy images of cells alone, cells with 1mg/mL spheres but no catalase, and cells with 1 mg/mL of spheres and catalase respectively (all in normoxic conditions – scale bar is 100 µm).*

1. C. L. Franco, J. Price and J. L. West, *Acta Biomaterialia*, 2011, 7, 3267-3276.
2. J. Oswald, S. Boxberger, B. Jørgensen, S. Feldmann, G. Ehninger, M. Bornhäuser and C. Werner, *Stem Cells*, 2004, 22, 377-384.
